# P01.23. Effects of unilateral facet fixation and facetectomy on muscle spindle responsiveness during simulated spinal manipulation

**DOI:** 10.1186/1472-6882-12-S1-P23

**Published:** 2012-06-12

**Authors:** W Reed, J Pickar

**Affiliations:** 1 Palmer College of Chiropractic, Davenport, USA

## Purpose

There is mounting evidence that identification of spinal joint hypo- and hypermobility along with correspondingly tailored manual therapy treatment approaches for these clinical subgroups can lead to more successful therapeutic outcomes. Subjects with spinal joint hypomobility had higher therapeutic success rates with spinal manipulation than subjects with spinal joint hypermobility (Fritz et al. Arch Phys Med Rehab 2005;86:1745-52). We were interested in whether changes in segmental mobility affect proprioceptive signaling from paraspinal muscles during spinal manipulation having thrust durations similar to those delivered clinically (75,100,150, 250ms).

## Methods

In the same anesthetized cat preparation, the L_5-6_ facet joints remained intact bilaterally, were screwed together unilaterally with a 10mm dental screw (fixation) or were completely removed unilaterally (facetectomy). All fixated/facetectomized preparations had at least a 4% change (increase/decrease) respectively in joint stiffness compared to the intact condition. Single unit activity from longissimus or multifidus muscle spindles was recorded from the L_6_ dorsal root. The L_6_ vertebra was manipulated in the posterior-to-anterior direction using a computer-controlled feedback motor coupled to the L_6_ spinous process through a pair of forceps.

## Results

Compared to baseline discharge, average mean instantaneous discharge frequency (ΔMIF) changed as follows [mean (SD) in impulses per second]:

**Table 1:** 

MANIPULATIVE THRUST DURATION:	75ms	100ms	150ms	250ms
Fixation (n=18):	102.8 (65.7)	85.5 (51.6)	61.0 (36.1)	46.6 (25.7)
Intact (n=18):	116.9 (60.4)	99.3 (55.1)	73.2 (34.5)	51.6 (22.6)
Facetectomy(n=8):	114.3 (54.5)	101.2 (68)	70.7 (36.1)	55.6 (27.1)

At each duration, compared with the intact condition ΔMIF during the manipulative thrust decreased with facet joint fixation but remained similar with facetectomy. In addition, the relationship between ΔMIF and changes in joint stiffness was not linear.

## Conclusion

The data suggest that segmental hypomobility but not segmental hypermobility alters the responsiveness of paraspinal muscle spindles during clinically relevant high velocity spinal manipulation. We speculate that difference in manipulation-evoked sensory input may relate to differences in spinal manipulation’s effects in subjects with hypomobility versus hypermobility.

